# Research on Registration Methods for Coupled Errors in Maneuvering Platforms

**DOI:** 10.3390/e27060607

**Published:** 2025-06-06

**Authors:** Qiang Li, Ruidong Liu, Yalei Liu, Zhenzhong Wei

**Affiliations:** 1 School of Instrumentation and Optoelectronics Engineering, Beihang University, Beijing 100083, China; zhenzhongwei@buaa.edu.cn; 2 No. 27 Research Institute of CETC, Zhengzhou 450047, China; 3 School of Artificial Intelligence, Henan University, Zhengzhou 450046, China; loner_d123@163.com (R.L.); liuyl3362@163.com (Y.L.)

**Keywords:** error calibration, mobile platform sensor registration, information entropy, coupled error estimation, mutual information coupling, pseudo-Kalman filter (PKF), joint estimation and registration

## Abstract

The performance limitations of single-sensor systems in target tracking have led to the widespread adoption of multi-sensor fusion, which improves accuracy through information complementarity and redundancy. However, on mobile platforms, dynamic changes in sensor attitude and position introduce coupled measurement and attitude errors, making accurate sensor registration particularly challenging. Most existing methods either treat these errors independently or rely on simplified assumptions, which limit their effectiveness in dynamic environments. To address this, we propose a novel joint error estimation and registration method based on a pseudo-Kalman filter (PKF). The PKF constructs pseudo-measurements by subtracting outputs from multiple sensors, projecting them into a bias space that is independent of the target’s state. A decoupling mechanism is introduced to distinguish between measurement and attitude error components, enabling accurate joint estimation in real time. In the shipborne environment, simulation experiments on pitch, yaw, and roll motions were conducted using two sensors. This method was compared with least squares (LS), maximum likelihood (ML), and the standard method based on PKF. The results show that the method based on PKF has a lower root mean square error (RMSE), a faster convergence speed, and better estimation accuracy and robustness. The proposed approach provides a practical and scalable solution for sensor registration in dynamic environments, particularly in maritime or aerial applications where coupled errors are prevalent.

## 1. Introduction

In modern target tracking applications, accurate and robust estimation is difficult to achieve with a single sensor due to performance limitations. To address this, this paper focuses on error registration in multi-sensor systems, especially for mobile platforms where measurement and attitude errors are coupled. The objective is to improve multi-sensor fusion accuracy through joint estimation of these errors using a pseudo-Kalman filter (PKF)-based approach.

Since it is difficult for a single sensor to achieve the tracking precision requirements in target tracking, combining data from several sensors is standard practice to improve performance [1]. By combining data from many sources, multi-sensor collaborative positioning can overcome equipment constraints and achieve information complementarity, improving tracking accuracy when compared to a single sensor [2]. Error calibration is an essential component of multi-source information fusion technology because data information from many sources has unique geographical, temporal, and state characteristics [3]. The reliability of the fused data will decline without error calibration, significantly lowering tracking accuracy. In recent years, advanced fusion strategies such as the robust unscented Kalman filter for decentralized INS/GNSS/CNS integration have been proposed to enhance estimation accuracy under complex navigation conditions [4]. Recent studies have also explored the application of deep learning models in improving the accuracy of target tracking and state estimation [5]. Additionally, the development of deep prediction networks based on covariance intersection fusion has shown promising results in sensor data analytics [6].

In the context of sensor registration, we encounter two scenarios: sensors mounted on fixed platforms and sensors installed on mobile platforms. For sensors on fixed platforms, the main source of measurement error is the equipment error of the sensors themselves [7]. For this type of problem, many relevant documents have studied the corresponding error registration algorithms. For example, the least squares (LS) method mentioned in [8], the general least squares (GLS) in [9], the maximum likelihood registration described in [10,11], and the real-time quality control in [12] are all good registration methods. However, in practical engineering applications, sensor measurements on mobile platforms are the common working scenarios. Unlike fixed platforms, the measurement references of sensors on mobile platforms undergo dynamic changes. When considering the implementation of error registration algorithms, it is necessary to take into account not only the measurement errors of the sensors themselves but also the attitude errors caused by the motion of the mobile platform [3]. Therefore, when considering the implementation of error registration algorithms, it is necessary to not only consider the measurement errors of the sensors themselves but also the attitude errors caused by the motion of the mobile platform. At present, a great deal of research has also been conducted on error registration for mobile platforms. The two-step registration method proposed in [13] first uses an extended Kalman filter to estimate the measurement errors of local sensors, then applies the unit quaternion method to calculate attitude and measurement errors, so as to achieve precise registration of sensor data. However, this method neglects the coupling between measurement errors and attitude errors, which may lead to a decrease in the system’s ability to respond to dynamic changes in complex environments, thereby affecting the performance of target tracking and state estimation. The maximum likelihood registration algorithm proposed in [14] estimates sensor measurement errors and target states through a batch processing approach to achieve precise registration of sensor data on mobile platforms. However, the performance of the algorithm significantly decreases when dealing with incomplete measurement models. An optimized bias model proposed in [15] aims to reduce the impact of attitude errors on measurements through simplified assumptions and quantitative analysis, to improve the estimation accuracy of radar measurement errors. However, when selecting the system state vector, the model actually neglects certain attitude errors, resulting in inaccurate measurement results. The mobile heterogeneous sensor registration algorithm based on maximum likelihood estimation proposed in [16] solves for coupled measurement errors and attitude errors. For incomplete measurement data, it uses linear minimum mean square error fusion technology for fusion estimation. However, it fails to perform joint estimation of measurement errors and attitude errors. The method proposed in [17] substitutes equivalent measurement errors for attitude errors, introducing the concept of equivalent measurement errors and treating attitude errors as part of the measurement errors to compensate for the filter. However, this approach may lead to algorithm designs that do not fully account for the dynamic characteristics of attitude errors, thereby limiting the adaptability of the system. In [18], the authors constructed an equivalent bias model and applied singular value decomposition (SVD) techniques to process measurement and attitude errors; however, the method also failed to perform joint estimation of measurement and attitude errors. In summary, existing research strategies for the two different types of errors, attitude errors and measurement errors, still employ independent registration or equivalent transformation fusion methods. These approaches fail to reflect the interaction between the two types of errors and thus cannot accurately estimate both errors. In addition, recent studies have proposed robust Kalman filtering techniques to address system bias and abnormal measurement errors under complex environments. For example, a hypothesis test-constrained robust Kalman filter was developed in [19] to mitigate the impact of abnormal measurements in INS/GNSS integration, and a robust unscented Kalman filter with measurement error detection was proposed in [20] for improving navigation accuracy in hypersonic vehicles.

This study suggests a coupled error joint estimation and registration approach for mobile platform error registration that is based on the pseudo-Kalman filter method (PKF) in order to address these problems. By using a decoupling technique, this method builds an accurate coupled error measurement model while accounting for the features of attitude and measurement errors. The pseudo-Kalman filter (PKF) estimates system deviations by constructing a pseudo-measurement model—independent of the target state—through projection of sensor observations, enabling joint error estimation without relying on a known target motion model [21]. At the same time, it can accomplish multi-sensor error registration for mobile platforms by utilizing the PKF to jointly estimate and register attitude faults and measurement errors.

## 2. System Description

**Figure 1:**
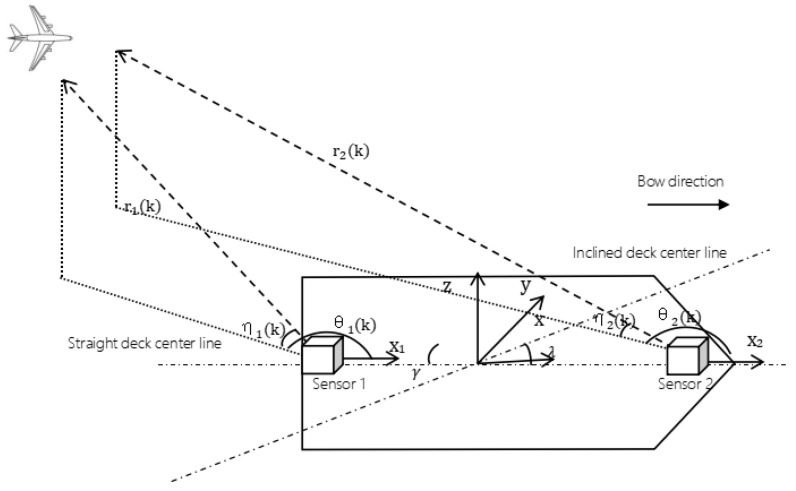
System description.

In the target tracking scenario, two sensors (Sensor 1 and Sensor 2) on a mobile platform independently measure an airborne moving target. Then, we create a precise target tracking trajectory using multi-source information fusion technology, which enables us to estimate and track the moving target. The situation is depicted in Figure 1.

The motion model of the target is

(1) X(k+1)=F(k)X(k)+V(k)

where X(k) is the target state vector at time step *k*, F(k) is the state transition matrix, and V(k) is the zero-mean white process noise.

For sensors *i*(*i* = 1, 2), the measurement equations are given by

(2) $$Z_{idp}\left(k\right)=\left[\begin{array}{c} r_{i}\left(k\right)\\ \theta_{i}\left(k\right)\\ \eta_{i}\left(k\right)\\ 0 \end{array}\right]=Z_{idp}\left(k\right)+b_{i}+w_{i}\left(k\right)=\left[\begin{array}{c} r_{i}^{\prime }\left(k\right)+b_{i}^{r}+w_{i}^{r}\\ \theta_{i}^{\prime }\left(k\right)+b_{i}^{\theta }+w_{i}^{\theta }\\ \eta_{i}^{\prime }\left(k\right)+b_{i}^{\eta }+w_{i}^{n}\\ 0 \end{array}\right]$$

where $Z_{idp}\left(k\right)$ denotes the independent measurement result of sensor *i* for the moving target, expressed in polar coordinates as $\left[r_{i}\left(k\right),\theta_{i}\left(k\right),\eta_{i}\left(k\right),0\right]$. Here, $r_{i}\left(k\right)$, $\theta_{i}\left(k\right)$, and $\eta_{i}\left(k\right)$ correspond to the target’s distance, azimuth angle, and elevation angle, respectively, measured by sensor *i* at time step *k*. $r_{i}^{\prime }\left(k\right)$, $\theta_{i}^{\prime }\left(k\right)$, and $\eta_{i}^{\prime }\left(k\right)$ represent the true measurement values without errors. $w_{i}\left(k\right)$ represents the noise bias of the sensor, which is assumed to be zero-mean white noise with a variance of $R(w_{i})$, assuming that measurement errors and measurement noises are independent of each other.

$b_{i}$ is the measurement error of the sensor *i*. In a mobile platform system, it is represented as a coupled error, which is related to measurement errors, attitude errors, and other perturbation factors. Assume that $b_{i}$ contains range error $b_{i}^{r}$, azimuth error $b_{i}^{\theta }$, elevation angle error $b_{i}^{\eta }$, roll angle error $b_{i}^{\phi }$, and pitch angle error $b_{i}^{\alpha }$, and assume they are constant additive errors described as

(3) $$b_{i}={\left[\left(b_{i}^{l}\right),{\left(b_{i}^{z}\right)}^{\prime }\right]}^{\prime }\left(i=1,2\right)$$

where $b_{i}^{l}={\left[b_{i}^{r},b_{i}^{\theta },b_{i}^{n},0\right]}^{\prime }$ represents the sensor’s measurement error, and $b_{i}^{z}={\left[b_{i}^{\phi },b_{i}^{\alpha },0,0\right]}^{\prime }$ represents the attitude error.

## 3. Registration Algorithm for Coupling Error of Mobile Platform

**Figure 2:**
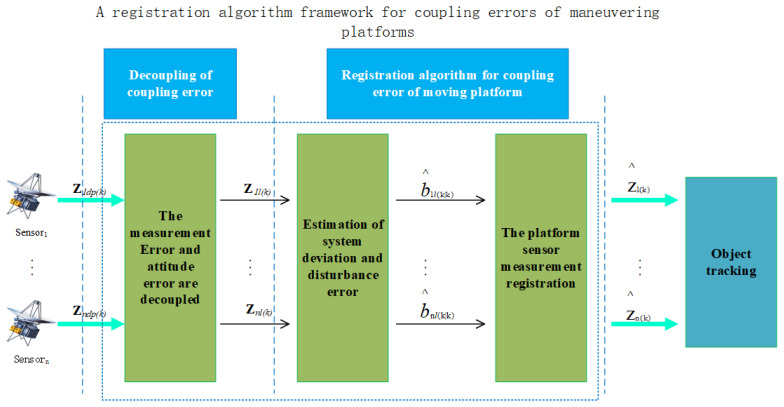
Framework of the algorithm.

In this section, we propose a registration algorithm for the coupling error of the maneuvering platform, which we use to first perform measurement error decoupling and measurement model construction for the coupling error of the previous section. Then the pseudo-measurement algorithm is used to jointly estimate the measurement error and attitude error, and finally, the estimated measurement error and attitude error are used to register the original measurements. The algorithm framework is shown in Figure 2.

### 3.1. Decoupling of Coupling Errors

The measurement error and attitude error are coupled in the measurement model $Z_{idp}\left(k\right)$. Due to such coupling, the estimation of the measurement error and attitude error are difficult to solve. Therefore, it is necessary to decouple the coupling error and construct a new measurement model. The specific steps of decoupling are as follows:

When tracking the target, the sensor measurement information is not only affected by the measurement error of the sensor device but also affected by the attitude error. Therefore, the measurement model of the sensor is as follows:

(4) $$Z_{il}\left(k\right)=A\left(\nu_{i}\left(k\right)+b_{i}^{z}\right)X\left(k\right)+b_{i}^{l}+w_{i}\left(k\right)$$

where $A\left(\nu_{i}\left(k\right)+b_{i}^{z}\right)$ is the state transition matrix containing the attitude information $v_{i}\left(k\right)=\left[\phi ,\alpha ,0,0\right]$ and attitude error $b_{i}^{z}$ of the platform.

(5) $$\begin{array}{cc} A(v_{i}\left(k\right)+b_{i}^{z}) & =A(\phi +\Delta \phi ,\alpha +\Delta \alpha )\\ \; & =A_{pitch}\left(\phi +\Delta \phi \right)A_{roll}\left(\alpha +\Delta \alpha \right)\\ \; & =\left[\begin{array}{cccc} \cos\left(\alpha +\Delta \alpha \right) & 0 & -\sin\left(\alpha +\Delta \alpha \right) & 0\\ \sin\left(\phi +\Delta \phi \right)\sin\left(\alpha +\Delta \alpha \right) & \cos\left(\phi +\Delta \phi \right) & \sin\left(\phi +\Delta \phi \right)\cos\left(\alpha +\Delta \alpha \right) & 0\\ \cos\left(\phi +\Delta \phi \right)\sin\left(\alpha +\Delta \alpha \right) & -\sin\left(\phi +\Delta \phi \right) & \cos\left(\phi +\Delta \phi \right)\cos\left(\alpha +\Delta \alpha \right) & 0\\ 0 & 0 & 0 & 1 \end{array}\right] \end{array}$$

The $A\left(\nu_{i}\left(k\right)+b_{i}^{z}\right)$ matrix is split into two matrices including attitude transition matrix B(k) and deviation component ΔM. The detailed decoupling steps are shown in Appendix A:

(6) $$A(v_{i}\left(k\right)-b_{i}^{z})=B\left(k\right)+\Delta M$$

The measurement equation of the sensor can be expressed as follows:

(7) $$Z_{il}\left(k\right)=B\left(k\right)X\left(k\right)+\left(C\left(k\right)b_{i}^{z}+b_{i}^{l}\right)+w_{i}\left(k\right)$$

Since $C\left(k\right)b_{i}^{z}=\Delta MX\left(k\right)$, we can find C(k) as follows:

(8) $$\begin{array}{cc} \; & C(k)\\ \; & =\left[\begin{array}{cccc} 0 & -\sin\alpha \cdot x(k)-\cos\alpha \cdot z(k) & 0 & 0\\ \cos\phi \sin\alpha \cdot x(k)-\sin\phi \cdot y(k)+\cos\phi \cos\alpha \cdot z(k) & \sin\phi \cos\alpha \cdot x(k)-\sin\phi \sin\alpha \cdot z(k) & 0 & 0\\ -\sin\phi \sin\alpha \cdot x(k)-\cos\phi \cdot y(k)-\sin\phi \cos\alpha \cdot z(k) & \cos\phi \cos\alpha \cdot x(k)-\cos\phi \sin\alpha \cdot z(k) & 0 & 0\\ 0 & 0 & 0 & 0 \end{array}\right] \end{array}$$

### 3.2. Registration of the Registration Algorithm Based on the Coupling Error of the Maneuvering Platform

#### 3.2.1. Joint Estimation of System Bias and Disturbance Error Based on Pseudo-Measurement

In the preceding part, we successfully separated the coupled disturbance in sensor measurement into independent measurement error and attitude error components. This section explains how to estimate and fix these separate error components online using the pseudo-Kalman Filter (PKF) technique for reliable sensor registration.

Two sensors observe the same target, and the two sensors on the moving platform are in the same measurement environment, which means that they are both impacted by the same attitude motion state and different attitude errors. The PKF algorithm performs online estimation of the sensor device measurement error and the attitude model error by projecting the space measurement information of the attitude disturbance error into the sensor system’s deviation space.

Firstly, the error of the sensor *i* measurement system is modeled. The state equation of the error can be expressed as follows:

(9) $$b_{i}\left(k+1\right)=F_{b}\left(k\right)b_{i}\left(k\right)+v_{b}^{i}\left(k\right)$$

The measurement equation including error can be expressed as follows:

(10) $$z_{b}^{i}\left(k\right)=B\left(k\right)X\left(k\right)+b_{i}\left(k+1\right)+w_{i}\left(k\right)=B\left(k\right)X\left(k\right)+\left(C\left(k\right)b_{i}^{z}+b_{i}^{l}\right)+w_{b}^{i}\left(k\right)$$

where $b_{i}\left(k\right)$ is the system error vector of the sensor, $F_{b}\left(k\right)$ is the system error state transition matrix, C(k) is the measurement matrix, $b_{i}^{z}$ is the attitude error of the ith sensor, $b_{i}^{l}$ is the device measurement error of the ith sensor, and $w_{b}^{i}\left(k\right)$ is the environmental noise of the sensor system.

Since two sensors observe the same target, that is, they have the same target state transition equation in the process of use, the common quantity can be subtracted from Equation (10) of the two sensors, so that the pseudo-measurement of the deviation vector can be defined as follows:

(11) $$\begin{array}{cc} z_{b}\left(k\right) & =z_{b}^{1}\left(k\right)-z_{b}^{2}\left(k\right)\\ \; & =B_{1}\left(k+1\right)F_{x}\left(k\right)X\left(k\right)+B_{1}\left(k+1\right)v_{x}\left(k\right)+b_{1}\left(k+1\right)+w_{1}\left(k+1\right)\\ \; & -[B_{2}\left(k+1\right)F_{x}\left(k\right)X\left(k\right)+B_{2}\left(k+1\right)v_{x}\left(k\right)+b_{2}\left(k+1\right)+w_{2}\left(k+1\right)] \end{array}$$

The two sensors are measured on the same carrier, so they are affected by the same state transition matrix, so b1=b2. The above formula is simplified as follows:

(12) $$\begin{array}{cc} z_{b}\left(k+1\right) & =z_{b}^{1}\left(k+1\right)-z_{b}^{2}\left(k+1\right)\\ \; & =C_{1}\left(k\right)b_{1}^{z}+b_{1}^{l}-\left(C_{2}\left(k\right)b_{2}^{z}+b_{2}^{l}\right)+w_{1}\left(k+1\right)-w_{2}\left(k+1\right) \end{array}$$

Compared with the previous method, which only uses the pseudo-Kalman filter to estimate the measurement errors of the sensor devices, this paper proposes an improved joint estimation method, which can not only estimate the measurement errors of the sensor devices, but also estimate the ship rocking model errors of the moving platform at the same time. This method integrates all error states into the same dimensional coordinate system for joint estimation, makes full use of the correlation between error states, and improves the estimation accuracy. In addition, the joint estimation method demonstrates clear advantages in computational efficiency and computational time, which can reduce the computational complexity and improve the real-time processing ability while ensuring the estimation accuracy, especially for efficient error compensation requirements in dynamic environments.

(13) $$z_{b}\left(k+1\right)=z_{b}^{1}\left(k+1\right)-z_{b}^{2}\left(k+1\right)=C_{b}\left(k+1\right)b\left(k+1\right)+W\left(k+1\right)$$

where

(14) $$C_{b}=\left[I\;C_{1}\left(k+1\right)\;-I\;-C_{2}\left(k+1\right)\right]$$

(15) $$b\left(k+1\right)=\left[\begin{array}{c} b_{1}^{l}\left(k+1\right)\\ b_{1}^{z}\left(k+1\right)\\ b_{2}^{l}\left(k+1\right)\\ b_{2}^{z}\left(k+1\right) \end{array}\right]$$

(16) $$W\left(k+1\right)=w_{1}\left(k+1\right)-w_{2}\left(k+1\right)$$

Equation (11) is the pseudo-measurement equation of the sensor system error.

The recursive process of the Kalman filter in the PKF algorithm is as follows:

Input: The prior estimate of the system bias $\hat{b}\left(k|k\right)$, the covariance matrix P(k), and the measurement $Z_{b}\left(k\right)$ at time *k*.

Output: The posterior estimate $\hat{b}\left(k+1|k+1\right)$ of the systematic bias at time k+1 and its covariance matrix P(k+1|k).

Calculate the system bias prediction $\hat{b}\left(k+1|k\right)$ and its covariance matrix P(k+1|k) at time k+1:

(17) $$\hat{b}\left(k+1|k\right)=F_{b}\hat{b}\left(k|k\right)$$

(18) $$P\left(k+1|k\right)=F_{b}P\left(k\right)F_{b}^{T}+Q_{b}\left(k+1\right)$$

Compute the innovation covariance matrix as follows:

(19) $$S\left(k+1\right)=C_{b}P\left(k+1|k\right)C_{b}^{T}+Q_{w}\left(k+1\right)$$

Calculate the gain matrix:

(20) $$K\left(k+1\right)=P\left(k+1|k\right)C_{b}^{T}S{\left(k+1\right)}^{-1}$$

Calculate the estimated system bias $\hat{b}\left(k+1|k+1\right)$ and its covariance matrix P(k+1) at time k+1 as follows:

(21) $$\hat{b}\left(k+1|k+1\right)=\hat{b}\left(k+1|k\right)+K\left(k+1\right)\left(z_{b}\left(k+1\right)-{\hat{z}}_{b}\left(k+1|k\right)\right)$$

(22) $$P\left(k+1\right)=P\left(k+1|k\right)-K\left(k+1\right)C_{b}P\left(k+1|k\right)$$

#### 3.2.2. Measurement Registration

To register the measurements, the following registration is performed based on Equations (21) and (22):

(23) $$\hat{z}\left(k+1\right)=z_{i}^{b}\left(k+1\right)-\hat{b}\left(k+1\right)$$

where $\hat{z}\left(k+1\right)$ is the measurement estimate at time k+1.

Through Equation (23), the measurement values with coupled measurement error and attitude error are registered, and then the Kalman filter is applied to the registered measurements to improve the accuracy of the target tracking system.

### 3.3. Algorithm Implementation

The pseudocode for the above algorithm is shown in Algorithm 1.
**Algorithm 1** Pseudocode of the algorithm**Input:** 
$\hat{b}\left(k|k\right),P\left(k\right),z_{b}\left(k\right)$
**Output:** 
$\hat{b}\left(k+1|k+1\right),P\left(k+1|k\right),\hat{z}\left(k+1\right)$
  1:Initialize parameter Settings.  2:**for** $k=1,\ldots \ldots ,K_{m}$ **do**.  3:    By (6), the state transition matrix $A(v_{i}\left(k\right)-b_{i}^{z})$ at time k can be decoupled into two matrices including attitude transition matrix B(k) and deviation component ΔM.  4:    By (8), the measurement matrix C(k) at time k can be obtained.  5:    By (17) and (18), the system deviation $\hat{b}\left(k+1|k\right)$ and the covariance matrix P(k+1|k) at time k+1 can be predicted.  6:    By (19) and (20), the innovation covariance matrix S(k+1) and gain matrix K(k+1) at time k+1 can be obtained.  7:    By (21) and (22), the system bias estimate $\hat{b}\left(k+1|k+1\right)$ and its covariance matrix P(k++1) at time k+1 can be obtained.  8:    By (23), the measurements can be registered to obtain $\hat{z}\left(k+1\right)$.  9:    Using the registered measurements for Kalman filtering or other processing……10:    Update variables $\hat{b}\left(k|k\right)$←$\hat{b}\left(k+1|k+1\right)$,P(k)←P(k+1|k) to prepare for the next iteration.11:**end for**12:**Note:** $K_{m}$ is the time to do large simulation.


## 4. Simulation and Analysis

### 4.1. Simulation Parameters

It is assumed that the target moves in a uniform linear motion in the air, and a physical scene is considered, that is, two sensors $S_{1}$ and $S_{2}$ on a ship detect the same target. The performance parameters of the two sensors are shown in Table 1 [22].

When the ship runs at a typical speed, the waves and wind act on the ship’s body, which will cause the pitch, roll, and yaw motion of the ship [23]. The three-axis motion of the ship can be described by the combination of the selected waves:

Yaw motion:

(24) $$\theta_{s}=0.25\sin\left(0.7t\right)+0.5\sin\left(0.1t\right)$$

Pitching motion:

(25) $$\phi_{s}=0.5\sin\left(0.6t\right)+0.3\sin\left(0.63t\right)+0.25$$

Rolling motion:

(26) $$\alpha_{s}=2.5\sin\left(0.5t\right)+3\sin\left(0.52t\right)+0.5$$

where t is time, (24)–(26) describe the change in three-axis motion with time.

The yawing is usually caused by wind, waves, and maneuvering; the amplitude and frequency are small, and the lateral displacement of the landing reference point is not enough to significantly affect the landing accuracy of the carrier aircraft. Therefore, in order to simplify the design and analysis of the control system, the impact of yawing on the landing ship can be ignored.

In the simulation scenario, the dynamic model of the target is described as

(27) $$X\left(k+1\right)=\left[\begin{array}{cccc} 1 & 0 & 0 & -vT\cos\gamma \cos\lambda \\ 0 & 1 & 0 & -vT\cos\gamma \sin\lambda \\ 0 & 0 & 1 & -vT\sin\gamma \\ 0 & 0 & 0 & 1 \end{array}\right]X\left(k\right)+V\left(k\right)$$

where γ is the angle between the target landing trajectory and the deck, λ is the angle between the oblique deck and the straight deck, and T=1 is the time interval of the discretization. $X\left(k\right)={\left[x\left(k\right)\;y\left(k\right)\;z\left(k\right)\;1\right]}^{\prime }$, (x(k),y(k),z(k)) denotes the position of the target in the Cartesian coordinate system, V(k)∼N(0,Q), and $Q=diag(Q_{x},Q_{y},Q_{z},0)$ is the process noise covariance, where $Q_{x}=\left[\begin{array}{cc} \frac{1}{3}T^{3} & \frac{1}{2}T^{2}\\ \frac{1}{2}T^{2} & T \end{array}\right]{\tilde{q}}_{x}$, $Q_{y}=\left[\begin{array}{cc} \frac{1}{3}T^{3} & \frac{1}{2}T^{2}\\ \frac{1}{2}T^{2} & T \end{array}\right]{\tilde{q}}_{y}$, $Q_{z}=\left[\begin{array}{cc} \frac{1}{3}T^{3} & \frac{1}{2}T^{2}\\ \frac{1}{2}T^{2} & T \end{array}\right]{\tilde{q}}_{z}$.

The measurement model is described as follows:

(28) $$Z_{il}\left(k\right)=A\left(v_{i}\left(k\right)+b_{i}^{z}\right)X\left(k\right)+b_{i}+W_{i}\left(k\right)$$

where $W_{i}\left(k\right)∼N\left(0,R\left(W_{i}\right)\right)$.

### 4.2. Performance Metrics

The performance measures of state estimate accuracy in this part are root mean square error (RMSE) and average RMSE (ARMSE), which are used to thoroughly assess the viability and superiority of the suggested method. Hellinger distance (HD), mean Hellinger distance (AHD), normalized estimation error (NEE), and mean NEE (ANEE) are the metrics used to evaluate the consistency of the state estimations.

First, RMSE and ARMSE are defined as follows:

(29) $$RMSE_{pos}=\sqrt{\frac{1}{MC}\sum_{mc=1}^{MC}\left({\left(p_{x,k}^{mc}-{\hat{p}}_{x,k}^{mc}\right)}^{2}+{\left(p_{y,k}^{mc}-{\hat{p}}_{y,k}^{mc}\right)}^{2}+{\left(p_{z,k}^{mc}-{\hat{p}}_{z,k}^{mc}\right)}^{2}\right)}$$

(30) $$ARMSE_{pos}=\sqrt{\frac{1}{K}\sum_{k=1}^{K}RMSE_{pos}{\left(k\right)}^{2}}$$

### 4.3. Results and Analysis

**Figure 3:**
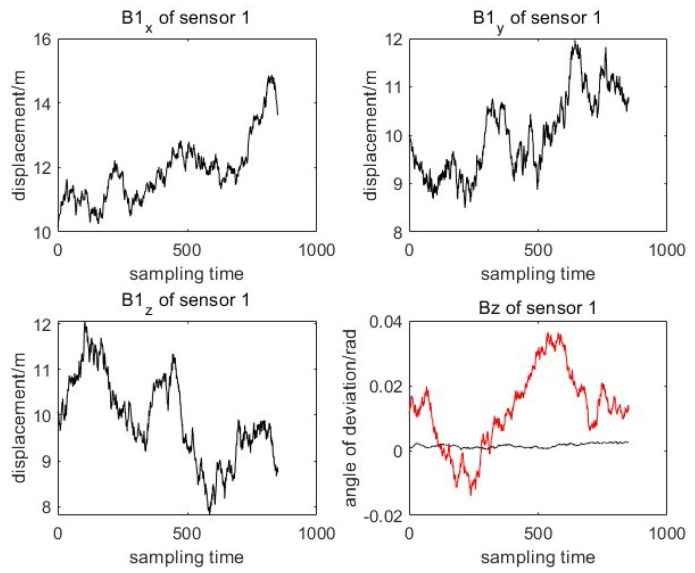
True value of coupling deviation for sensor 1.

**Figure 4:**
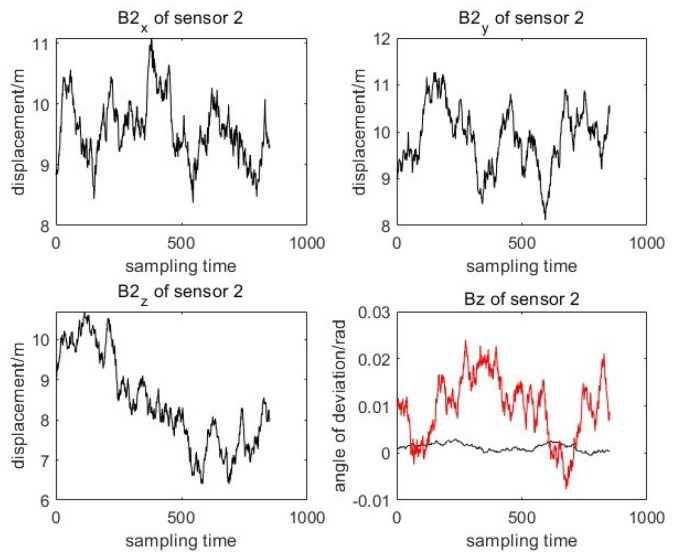
True value of coupling deviation for sensor 2.

Two sensors $1(S_{1})$ and $2(S_{2})$ with different system deviations are used to measure the moving target without registration. The changes in sensor system deviation and attitude error are shown in Figure 3 and Figure 4. It can be seen from the system deviation diagram of sensor 1 and sensor 2 that the sensor system deviation is not constant, and it will change slightly due to external noise interference.

**Figure 5:**
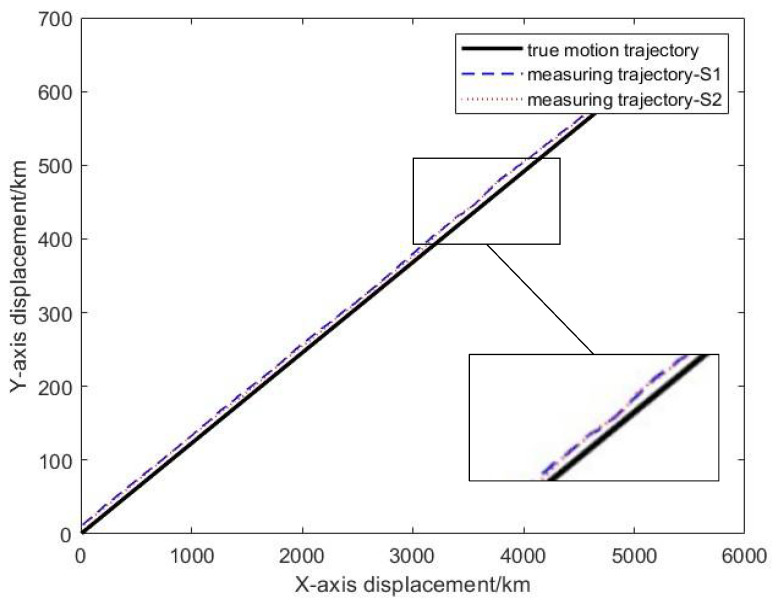
Measurement trajectory of the sensor under the influence of coupling error.

Considering the influence of sensor coupling deviation, the measurement results of the moving target have disturbance deviation. Therefore, under the above simulation background, the measured trajectory of the target aircraft is shown in Figure 5.

**Figure 6:**
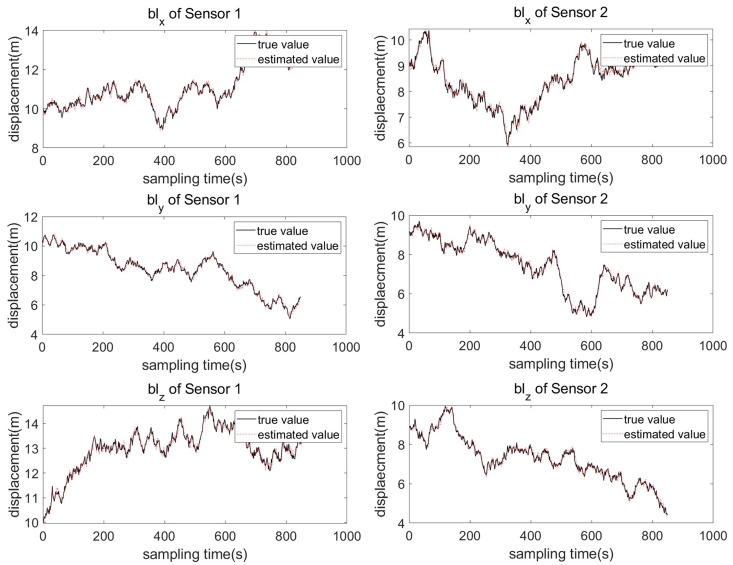
Comparison of the true and estimated system deviation of sensor bl.

**Figure 7:**
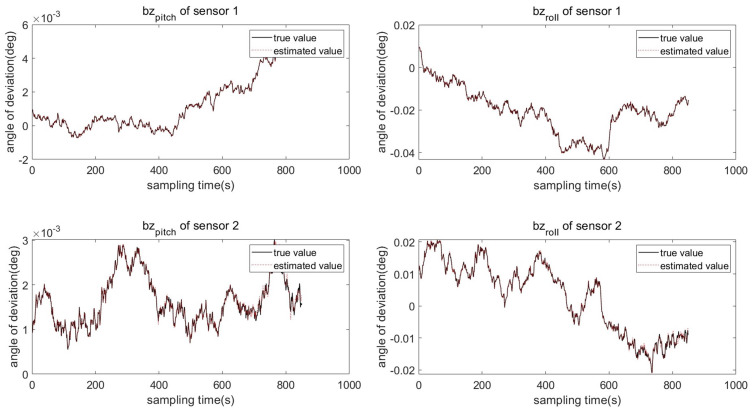
Comparison of the true and estimated system deviation of sensor bz.

The target trajectory is measured by two sensors without registration, and the measurement results are affected by many factors, such as sensor system bias, attitude error, and measurement noise. It can be seen from the above figure that the measured trajectory without sensor deviation registration is quite different from the real trajectory, especially in the part affected by the disturbance, the measured trajectory deviates significantly. To make the measured trajectory reflect the real trajectory of the target accurately, the PKF-JE algorithm proposed in this chapter is used to estimate the deviation of the sensor system, and then the measured trajectory registration is realized. Figure 6 and Figure 7 represent the comparison plots of the estimated values of the sensor system bias and the true values at each sampling time using the PKF algorithm proposed in this chapter. It can be seen from the figure that except for the large difference between the estimated value and the true value in the first few sampling moments, the difference between the two curves is small in the whole estimation process after that. That is, the difference between the estimated value and the true value of the system deviation is very small.

**Figure 8:**
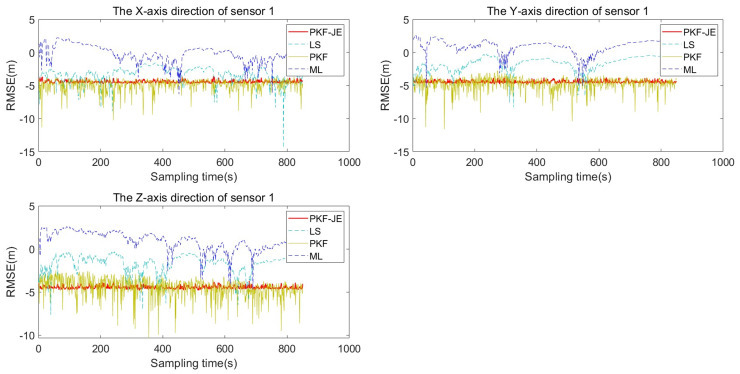
Comparison of RMSE log curves for sensor 1.

**Figure 9:**
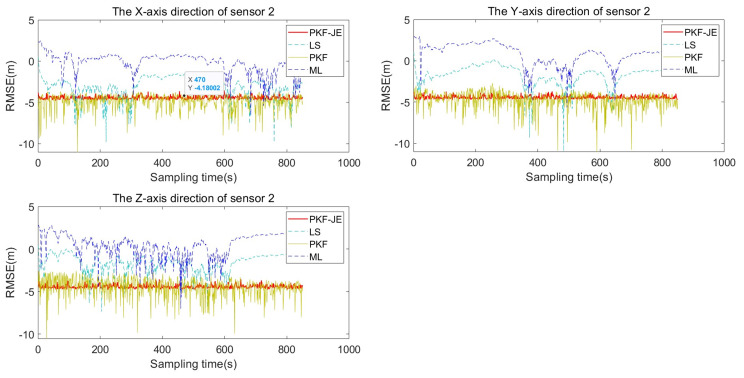
Comparison of RMSE log curves for sensor 2.

To prove the superiority of this algorithm, this section uses the LS algorithm and PKF algorithm to compare and analyze the proposed PKF-JE algorithm. Firstly, Monte Carlo simulations of the three algorithms were carried out independently. To highlight the differences and facilitate the analysis, the root mean square deviation was logarithmically processed, and the RMSE logarithmic curve after 100 Monte Carlo simulations is shown in Figure 8 and Figure 9. It is easy to see from the figures that the PKF-JE algorithm has the best effect among the three algorithms, and the logarithm curve of RMSE of LS and PKF algorithm obviously jumps.

The average values of the root mean square deviations (RMSDs) for the four algorithms under perturbation are presented in Table 2. Taking the Y-axis deviation of sensor 1 as an example, the PKF-JE algorithm yields an average RMSD of 0.0122, which is significantly lower than that of the PKF (0.0162), LS (0.2413), and ML (0.4351) algorithms. As shown in Figure 8 and Figure 9, the proposed PKF-JE algorithm exhibits the fastest convergence rate and consistently maintains the highest estimation accuracy throughout the simulation period. Overall, PKF-JE outperforms the traditional PKF, LS, and ML methods in all three spatial directions for both sensors, demonstrating greater robustness and precision in the presence of perturbations.

## 5. Conclusions

The average root mean square deviation values and the comparison of convergence speed and estimation accuracy provide a comprehensive assessment of the four algorithms’ performances. With the minimum average RMSE value of 0.0122 for the Y-axis deviation of sensor 1, the PKF-JE algorithm demonstrates superior estimation accuracy, significantly outperforming the LS algorithm (0.2413), the PKF algorithm (0.0162), and the ML algorithm (0.4351). Furthermore, throughout the simulation, the proposed PKF-JE method consistently achieves higher estimation accuracy and the fastest convergence across all system deviations. Compared to the conventional PKF, LS, and ML algorithms, PKF-JE exhibits noticeably better overall performance, making it a more reliable and efficient choice for system deviation estimation under perturbation.

In summary, this study advances the field by offering a more precise and faster-converging method for system deviation estimation, which is crucial for enhancing the reliability of multi-sensor systems. While the proposed PKF-JE algorithm demonstrates significant advantages, its performance may be influenced by specific system configurations and environmental conditions, indicating areas for further refinement. Future research could explore the algorithm’s adaptability to more complex, dynamic environments and investigate methods to optimize its performance across a broader range of applications.

## Figures and Tables

**Table 1:** Sensor parameters.

Sensor	$R(W_{i})$	$b_{i}^{l}$	$b_{i}^{z}$
$S_{1}$	0.01	0.001	1
$S_{2}$	0.013	0.0013	0.95

**Table 2:** Average RMS deviations of the three algorithms under the influence of perturbation.

	$S_{1}¯X$	$S_{1}¯Y$	$S_{1}¯Z$	$S_{2}¯X$	$S_{2}¯Y$	$S_{2}¯Z$
PKF-JE	0.0123	0.0122	0.0123	0.0123	0.0127	0.0123
PKF	0.0126	0.0162	0.0123	0.0130	0.0168	0.0290
LS	0.0852	0.2413	0.2534	0.0891	0.3414	0.4340
ML	0.1131	0.4351	0.3752	0.1103	0.5135	0.6342

## Data Availability

No new data were created or analyzed in this study. Data sharing is not applicable to this article.

## Appendix A

(A1)
$$\begin{array}{cc} & A(v_{i}\left(k\right)-b_{i}^{z})\\ & =M\\ & =\left[\begin{array}{cccc} \cos\left(\alpha +\Delta \alpha \right) & 0 & -\sin\left(\alpha +\Delta \alpha \right) & 0\\ \sin\left(\phi +\Delta \phi \right)\sin\left(\alpha +\Delta \alpha \right) & \cos\left(\phi +\Delta \phi \right) & \sin\left(\phi +\Delta \phi \right)\cos\left(\alpha +\Delta \alpha \right) & 0\\ \cos\left(\phi +\Delta \phi \right)\sin\left(\alpha +\Delta \alpha \right) & -\sin\left(\phi +\Delta \phi \right) & \cos\left(\phi +\Delta \phi \right)\cos\left(\alpha +\Delta \alpha \right) & 0\\ 0 & 0 & 0 & 1 \end{array}\right] \end{array}$$

Take $A{\left(v_{i}\left(k\right)-b_{i}^{z}\right)}_{21}$ as an example to obtain the elements in $A(v_{i}\left(k\right)-b_{i}^{z})$ by trigonometric decoupling through the sum angle formula:

(A2) $$\begin{array}{cc} \sin(\phi +\Delta \phi )\sin(\alpha +\Delta \alpha )= & \sin\phi \sin\alpha \cos\Delta \phi \cos\Delta \alpha +\sin\phi \cos\alpha \cos\Delta \phi \sin\Delta \alpha \\ & +\cos\phi \sin\alpha \sin\Delta \phi \cos\Delta \alpha +\cos\phi \cos\alpha \sin\Delta \phi \sin\Delta \alpha \; \end{array}$$

Since Δα and Δϕ are relatively small quantities, we can approximate cosΔa≈1, sinΔa≈Δα and ignore the higher-order term Δϕ, Δα, which is obtained as follows:

(A3) sin(ϕ+Δϕ)sin(α+Δα)≈sinϕsinα+Δα(sinϕcosα)+Δϕ(cosϕsinα)

Decoupling the other elements of *M* in the same way gives us

(A4) $$\begin{array}{cc} & A(v_{i}\left(k\right)-b_{i}^{z})\\ & =\left[\begin{array}{cccc} \cos\alpha -\sin\alpha \Delta \alpha & 0 & -\sin\alpha -\cos\alpha \Delta \alpha & 0\\ \sin\phi \sin\alpha +\cos\phi \Delta \phi \sin\alpha +\sin\phi \cos\alpha \Delta \alpha & \cos\phi -\sin\phi \Delta \phi & \sin\phi \cos\alpha +\cos\phi \Delta \phi \cos\alpha -\sin\phi \sin\alpha \Delta \alpha & 0\\ \cos\phi \sin\alpha -\sin\phi \Delta \phi \sin\alpha +\cos\phi \cos\alpha \Delta \alpha & -\sin\phi -\cos\phi \Delta \phi & \cos\phi \cos\alpha -\sin\phi \Delta \phi \cos\alpha -\cos\phi \sin\alpha \Delta \alpha & 0\\ 0 & 0 & 0 & 1 \end{array}\right] \end{array}$$

Split the $A(v_{i}\left(k\right)-b_{i}^{z})$ matrix into two matrices including disturbance and deviation:

(A5) $$A(v_{i}\left(k\right)-b_{i}^{z})=B\left(k\right)+\Delta M$$

where

(A6) $$B\left(k\right)=\left[\begin{array}{cccc} \cos\alpha & 0 & -\sin\alpha & 0\\ \sin\phi \sin\alpha +\sin\phi \cos\alpha & \cos\phi & \sin\phi \cos\alpha +\cos\phi \cos\alpha & 0\\ \cos\phi \sin\alpha +\cos\phi \cos\alpha & -\sin\phi & \cos\phi \cos\alpha & 0\\ 0 & 0 & 0 & 1 \end{array}\right]$$

(A7) $$\Delta M=\left[\begin{array}{cccc} -\sin\alpha \Delta \alpha & 0 & -\cos\alpha \Delta \alpha & 0\\ \cos\phi \Delta \phi \sin\alpha +\sin\phi \cos\alpha \Delta \alpha & -\sin\phi \Delta \phi & \cos\phi \Delta \phi \cos\alpha -\sin\phi \sin\alpha \Delta \alpha & 0\\ -\sin\phi \Delta \phi \sin\alpha +\cos\phi \cos\alpha \Delta \alpha & -\cos\phi \Delta \phi & -\sin\phi \Delta \phi \cos\alpha -\cos\phi \sin\alpha \Delta \alpha & 0\\ 0 & 0 & 0 & 0 \end{array}\right]$$
